# An enhanced bioluminescence-based Annexin V probe for apoptosis detection *in vitro* and *in vivo*

**DOI:** 10.1038/cddis.2017.141

**Published:** 2017-05-25

**Authors:** Trajen Head, Peter Dau, Stephanie Duffort, Pirouz Daftarian, Pratibha M Joshi, Roberto Vazquez-Padron, Sapna K Deo, Sylvia Daunert

**Affiliations:** 1 Department of Biochemistry and Molecular Biology, University of Miami Miller School of Medicine, Miami, FL 33136, USA; 2 Department of Ophthalmology, University of Miami Miller School of Medicine, Miami, FL 33136, USA; 3 NGM Biopharmaceuticals, Inc., South San Francisco, CA 94080, USA; 4 Department of Surgery, University of Miami Miller School of Medicine, Miami, FL 33136, USA

## Abstract

The process of controlled cellular death known as apoptosis has an important central role not only in normal homeostatic maintenance of tissues, but also in numerous diseases such as cancer, neurodegenerative, autoimmune, and cardiovascular diseases. As a result, new technologies with the capability to selectively detect apoptotic cells represent a central focus of research for the study of these conditions. We have developed a new biosensor for the detection of apoptotic cells, incorporating the targeted selectivity for apoptotic cells from Annexin V with the sensitivity of bioluminescence signal generation from a serum-stable mutant of *Renilla* luciferase (RLuc8). Our data presents a complete characterization of the structural and biochemical properties of this new Annexin-*Renilla* fusion protein (ArFP) construct, as well as a validation of its ability to detect apoptosis *in vitro*. Moreover, this work represents the first report of a bioluminescent Annexin V apoptosis sensor utilized *in vivo*. With this new construct, we examine apoptosis within disease-relevant animal models of surgery-induced ischemia/reperfusion, corneal injury, and retinal cell death as a model of age-related macular degeneration. In each of these experiments, we demonstrate successful application of the ArFP construct for detection and bioluminescence imaging of apoptosis within each disease or treatment model. ArFP represents an important new tool in the continuously growing kit of technologies for apoptosis detection, and our results from both *in vitro* and *in vivo* experiments suggest a diverse range of potential clinically relevant applications including cancer therapeutic screening and efficacy analysis, atherosclerosis and cardiovascular disease detection, and the monitoring of any number of other conditions in which apoptosis has a central role.

The presence of controlled cell death has been identified in a diverse range of cell and tissue types as far back as the mid-nineteenth century.^1, 2^ However, it was not until the introduction of the unifying term ‘apoptosis’ in the 1970’s that the importance of this ubiquitous process was rigorously examined.^3^ Since that time, it has become apparent that apoptosis is not only crucial for normal homeostatic cell maintenance within whole tissues, but is also directly involved in a number of pathologies including cancer, neurodegenerative diseases (Alzheimer’s and Parkinson’s), cardiovascular disease, and autoimmune diseases.^4, 5, 6, 7^ Because both apoptosis and defects in apoptotic pathways are known to be essential components of these issues, a critical focus of research in this field has been centered on the development of techniques and platforms capable of selectively detecting apoptotic cells.

Although the numerous pathways of apoptosis initiation are varied and diverse, most coalesce at the activation of caspases (Figure 1). Once activated, these proteases are directly responsible for the biochemical and morphological changes that define the apoptotic process, including nuclear condensation, cellular blebbing, and – importantly – the exposure of the membrane phospholipid phosphatidylserine (PS) on the surface of the cell.^8, 9, 10, 11, 12^ Herein, we describe the design, development, and implementation of a bioluminescent protein biosensor that specifically targets this morphological change, requires no post-expression modification, and provides an effective means to detect apoptotic cells both *in vitro* and *in vivo*.

Annexin V is an endogenous human protein (encoded by the ANXA5 gene) that binds specifically to PS with a high affinity (~10^−9^ M) (Supplementary Figure S1).^13, 14^ Previous work describes Annexin V modified with either fluorescent^15, 16, 17, 18^ or radiolabel^19, 20, 21^ reporters and used for the detection of apoptosis, but presents numerous challenges. Detection approaches utilizing fluorescence, specifically in the context of detection within a biological sample, inherently suffer from issues of high background signal and low sensitivity based on the prevalence of numerous fluorescent species found in biological samples.^22^ Isotopic labeling carries restrictive regulations on handling, waste disposal, and health effects, whereas both of these approaches exhibit a high degree of variability in consistency of reagent preparation, presenting another major hurdle. Although the use of these detection methods is less than ideal, the *in vivo* detection of apoptosis still represents an important avenue of research, and has been employed to monitor a range of events such as cardiac allograft transplant stability and chemotherapeutic efficacy.^23, 24^ To that end, the use of bioluminescence presents an opportunity to address the inherent challenges of apoptosis detection presented by these other methods.

*Renilla* luciferase (RLuc) is an enzyme natively expressed by the sea pansy *Renilla reniformis* that oxidatively decarboxylates its substrate coelenterazine to generate bioluminescence (Supplementary Figure S1).^25, 26^ Detection of this type of emission generally results in lower background and higher signal-to-noise ratios, providing a means of detection that is more sensitive than fluorescence, especially in the context of biological samples.^22, 27^ However, it has been shown that this wild-type RLuc is quickly rendered inactive in the presence of murine serum,^28^ strictly limiting previous work with a *Renilla* luciferase-labeled Annexin V probe to applications *in vitro*.^29, 30^ Protein engineering of the wild-type RLuc protein has yield a luciferase variant (RLuc8) that exhibits a 200-fold increase in serum stability, as well as a 4-fold increase in light output.^28^ This affords a unique opportunity to couple its use with relevant *in vivo* disease models to detect apoptosis, and analyze the role which apoptosis has within them.

We have utilized this RLuc8 mutant to generate a chimeric bioluminescence-based Annexin V apoptosis detection construct. We first analyze the structural and biochemical properties of this Annexin V-*Renilla* luciferase fusion protein (also referred to as ArFP) to verify that the construct retains the ability to both bind to PS while also remaining capable of bioluminescent emission. As shown in Figure 1, under apoptotic conditions when PS is no longer sequestered on the inner-face of the cell membrane, ArFP will bind to the surface of the apoptotic cells, allowing for detection via bioluminescence generation from addition of the substrate coelenterazine. Importantly, we demonstrate that, in addition to functioning as a specific sensor for apoptosis *in vitro*, this construct also allows for the bioluminescence imaging of apoptosis *in vivo*. With this functionality, we are able to examine the involvement of apoptosis within the context of entire tissues in processes such as surgery-induced vascular apoptosis and age-related macular degeneration (AMD), providing a new means to observe and utilize these and other models of disease-specific apoptosis.

## Results

This research introduces a new and important expansion to the currently available toolkit of Annexin V-based detection systems for the specific identification of apoptosis. Specifically, herein we report the cloning and production of a chimeric fusion protein of Annexin V and RLuc8 (ArFP), and performed a comprehensive analysis of both the physical and biochemical characteristics of this fusion. We also demonstrated that the biosensor is capable of apoptosis detection *in vitro*. Moreover, this study represents the first description of a bioluminescent Annexin V apoptosis sensor successfully applied *in vivo*, providing new means to assess clinically relevant disease models involving apoptosis.

### Design, expression, and characterization of ArFP

**Figure 2:**
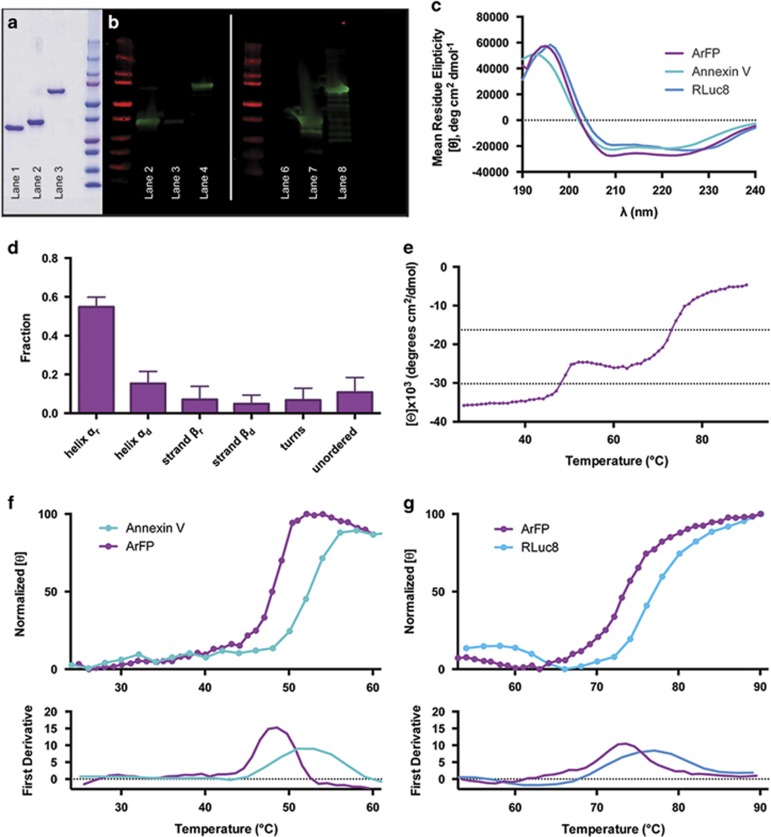
(**a**) SDS-PAGE analysis of components and fusion protein (lane 1: Annexin V, lane 2: RLuc8, lane 3: ArFP, lane 4: Precision Plus Protein Ladder (Bio-Rad)). (**b**) Western blot analysis of components and fusion protein identified using anti-Annexin V antibody (lane 1: Precision Plus Protein Ladder, lane 2: Annexin V, lane 3: RLuc8, lane 4: ArFPP) or anti-*Renilla* luciferase antibody (lane 5: Precision Plus Protein Ladder, lane 6: Annexin V, lane 7: RLuc8, lane 8: ArFP). (**c**) Far-UV CD spectra of ArFP (purple), Annexin V (teal), and RLuc8 (blue). (**d**) Protein 2° structure assignment (CDSSTR analysis via Dichroweb) for ArFP. (**e**) Thermostability analysis (ellipticity at 222 nm) of ArFP. (**f**) Comparison of Annexin V and ArFP thermostability (upper) and first derivative analysis of curves (lower). (**g**) Comparison of RLuc8 and ArFP thermostability (upper) and first derivative analysis of curves (lower)

Genes for both Annexin V and RLuc8 were cloned out of their respective plasmids, and combined in a single fusion gene which was inserted into the pET-30 Xa/LIC vector to generate pET-30/ArFP (see Materials and Methods, Supplementary Figures S2 and S3). Following transformation of this vector, ArFP was expressed in *E. coli* as soluble protein, and isolated using immobilized metal affinity chromatography. Before this novel bioluminescent Annexin V-based biosensor could be utilized in apoptosis detection, both the physical and biochemical properties were characterized. Analysis by SDS-PAGE indicated that isolated ArFP had a purity exceeding 95% and exhibited an approximate size of 73 kDa, whereas purified samples of Annexin V and RLuc8 demonstrated similar purities with sizes of 36 kDa and 37 kDa, respectively (Figure 2a). Identification of this isolated construct by antibodies against both Annexin V and RLuc8 via western blot analysis (Figure 2b) supports the chimeric incorporation of both of these components into a single fusion, requiring no post-expression workup or modification. Similarities of the circular dichroism (CD) spectra for each of the three proteins in regions corresponding to secondary structure contributions also suggest that the fusion of Annexin V and RLuc8 has not compromised the individual secondary structure integrity of the proteins (Figure 2c). The far-UV CD spectrum for ArFP was further analyzed by the CDSSTR analysis package, generating secondary structure assignments for the fusion (Figure 2d). The presence of predominantly *α*-helical structure with minor *β*-sheet contribution in the fusion is in agreement with the known crystal structures of RLuc8 and Annexin V, the latter consisting nearly exclusively of *α*-helices (Supplementary Figure S1). In addition, the denaturation temperature (*T*_m_) of the fusion protein was determined, and exhibited two distinct transition phases at ~48 °C and 73 °C (Figure 2e). Comparison of temperature-relevant sections of this curve to thermostability measurements made for Annexin V and RLuc8 indicate that each transition roughly corresponds with each component of the fusion (Figures 2f and g). Taken together, these results from SDS-PAGE, western blot, and CD spectra analysis indicate that ArFP presents a combination of the structural characteristics of both Annexin V and RLuc8 with no need for post-expression modifications. Unlike previous Annexin V reporters relying on fluorescence or isotopic labeling, ArFP maintains a strict stoichiometric ratio of Annexin V and RLuc8 with no batch-to-batch variability. In addition, the CD thermostability observed in the spectra of the fusion protein suggests that the two components of the fusion remain largely independent of each other, and should retain both PS-binding and bioluminescence activities at physiologically relevant temperatures.

### Activity characterization of ArFP

**Figure 3:**
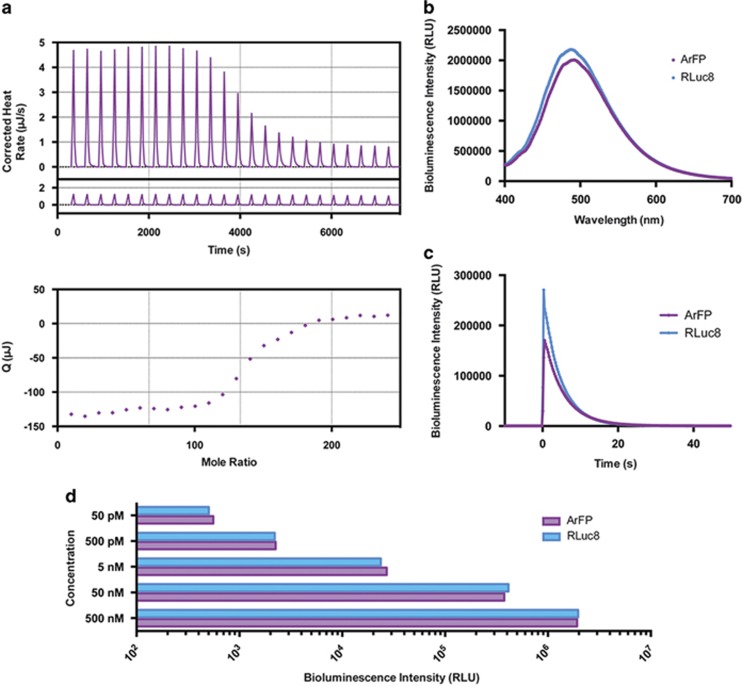
(**a**) Top: ITC data from the titration of 30 *μ*M ArFP with lipid vesicles composed of 60:40 mixture of POPC:POPS in the presence of 0.75 mM Ca^2+^ at 18 °C. Middle: Heat of dilution (control/baseline). Bottom: Integrated heats of binding as a function of ligand:protein mole ratio. (**b**) Bioluminescence spectra from 400  to 700 nm resulting from addition of coelenterazine substrate to ArFP (purple) or RLuc8 (blue). (**c**) Kinetic bioluminescence data resulting from injection of coelenterazine substrate to ArFP (purple) or RLuc8 (blue) (250 integrations at 0.24 s/each). (**d**) Concentration-dependent bioluminescence output comparison of ArFP (purple) or RLuc8 (blue) after injection of coelenterazine substrate (10 s integration). All points are the mean of three measurements±one S.D. Error bars that are not visible are obstructed by the data point

Analysis of the protein activity of ArFP began with an investigation into its ability to bind PS. Isothermal titration calorimetry (ITC) was used to calculate the binding affinity of ArFP for PS exposed on the surface of lipid vesicles (Figure 3a). The dissociation constant (*K*_D_) of 20.7 *μ*M calculated for ArFP closely matched the previously reported value of 13 *μ*M for native Annexin V, and suggests that ArFP will interact with exposed PS on cellular surfaces in an analogous manner (Table 1).^31^ Comparatively, the bioluminescence characteristics of ArFP were also found to be highly similar to those of native RLuc8 (Figures 3b–d). Minor variations were observed from RLuc8 to ArFP, including a red-shift in wavelength of peak emission, and a slight increase in emission half-life (Table 1). However, total luminescence output measured across a range of protein concentrations from 50 pM to 500 nM resulted in no statistical difference at any tested concentration (Figure 3d). On the basis of the results of this characterization of both the physical and biochemical properties of ArFP, we were confident that the fusion protein would be capable of binding to surface-exposed PS while also generating a detectable bioluminescent signal either *in vitro* or *in vivo*.

### Apoptosis detection *in vitro*

**Figure 4:**
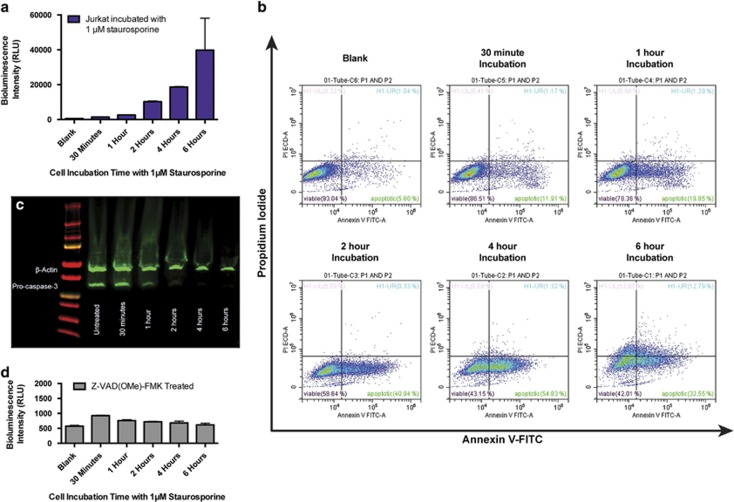
(**a**) Bioluminescence response of ArFP incubated with Jurkat cells after treatment with 1 *μ*M staurosporine for various times. Bioluminescence intensity reported as the mean of three measurements±one S.D. Error bars that are not visible are obstructed by the data point. (**b**) Flow cytometry analysis of Jurkat cells following treatment with 1 *μ*M staurosporine for various times and dual stained with Annexin-FITC (FL1, 525/40 band pass filter) and the vital dye PI (FL11, 610/20 band pass filter) (Data acquisition: 2 × 10^4^ events per sample). (**c**) Western blot analysis of Jurkat cell lysates following treatment with 1 *μ*M staurosporine for various times. (**d**) Bioluminescence response of ArFP incubated with Jurkat cells treated with 25 *μ*M caspase inhibitor Z-VAD(OMe)-FMK followed by treatment with 1 *μ*M staurosporine for various times. Bioluminescence intensity reported as the mean of three measurements±one S.D. Error bars that are not visible are obstructed by the data point

Having demonstrated that the physical and biochemical properties of both Annexin V and RLuc8 had been successfully incorporated into ArFP, we tested the ability of this construct to detect apoptosis *in vitro*. ArFP was added to Jurkat (human T lymphocyte) cells that had been treated with the known apoptosis inducer staurosporine.^32, 33^ Bioluminescence intensities were then measured following the addition of the substrate coelenterazine, and as shown, exhibit increasing luminescence intensities as the staurosporine incubation time increases (Figure 4a). These observations were validated through the use of flow cytometric analysis, in which cells treated with staurosporine were subsequently stained using fluorescently labeled Annexin V (Annexin-FITC) and the vital dye propidium iodide (PI) (Figure 4b). As observed, an increase in staurosporine incubation results in an increase in apoptotic cells (Annexin-FITC^+^/PI^−^), whereas loss of membrane integrity (Annexin-FITC^−^/PI^+^ or Annexin-FITC^+^/PI^+^) wasn’t observed until the 6-h time point. The activation of caspase-3 represents another reliable indicator of apoptosis induction as it serves as a convergence point for many different apoptotic pathways. This activation involves processing of caspase-3’s pro-enzyme form to its active proteolytic form, and is known to be responsible for the display of PS in apoptotic cells once activated.^8, 34^ Western blot analysis of staurosporine treated and control Jurkat cell lysates clearly demonstrate a reduction in the levels of pro-caspase-3 dependent on staurosporine incubation duration, suggesting the liberation of activated caspase-3 (Figure 4c). To investigate ArFP’s specificity in targeting PS exposed via this caspase activation during apoptosis, Jurkat cells were pre-incubated with the cell permeable, pan-caspase inhibitor Z-VAD(OMe)-FMK prior to staurosporine treatment. Following incubation with both the apoptosis inducer and the caspase inhibitor, ArFP was added to the treated cells, but the resulting bioluminescence measurements showed no significant intensity increase with respect to incubation time (Figure 4d). These data demonstrate that the induction of apoptosis and the activation of caspases within the cell are responsible for an increase in the number of bound ArFP constructs, directly resulting in an increase in bioluminescence signal intensity. In addition, bioluminescence-based apoptosis detection of staurosporine-treated cells was performed using a second cell line, namely, Caco-2 human epithelial colorectal adenocarcinoma cells (Supplementary Information; Supplementary Figure S5). Finally, as a confirmation of staurosporine impact on the health of these cells, the cellular metabolic activity was examined with respect to staurosporine incubation duration for both Caco-2 (Supplementary Figure S5) and Jurkat (Supplementary Figure S6) cells. These experiments confirmed that ArFP is capable of *in vitro* apoptosis detection by the generation of bioluminescence signal in a rapid and highly specific manner.

### Apoptosis detection *ex vivo*

**Figure 5:**
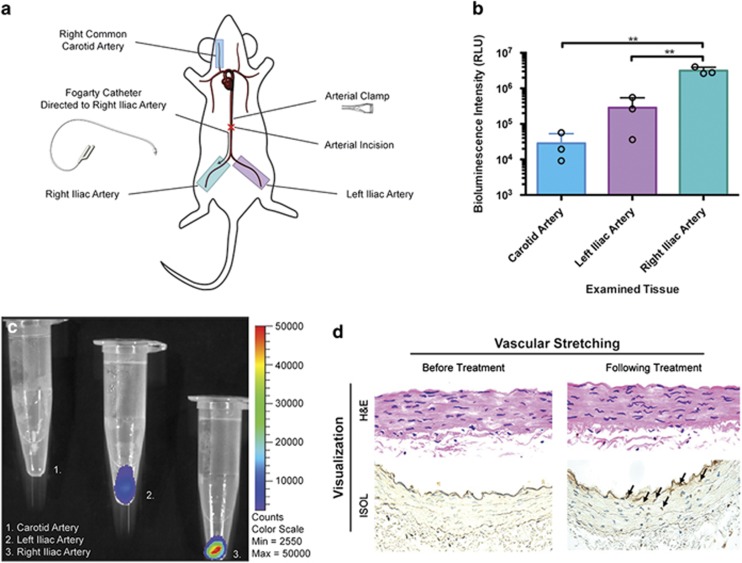
(**a**) Diagram of operational procedure, illustrating position of treated and collected tissues (teal: right iliac/treated condition, purple: left iliac/reperfusion control, blue: carotid/healthy control). (**b**) Luminescence comparisons of treated (teal) and untreated (purple) iliac arteries as well as the carotid (blue) artery as a healthy control, performed in triplicate. (**c**) IVIS image of the carotid (1), left iliac (2), and right iliac (3) arteries after treatment. (**d**) Histological analysis of treated vascular tissue indicating the emergence of apoptotic cells following treatment

**Figure 6:**
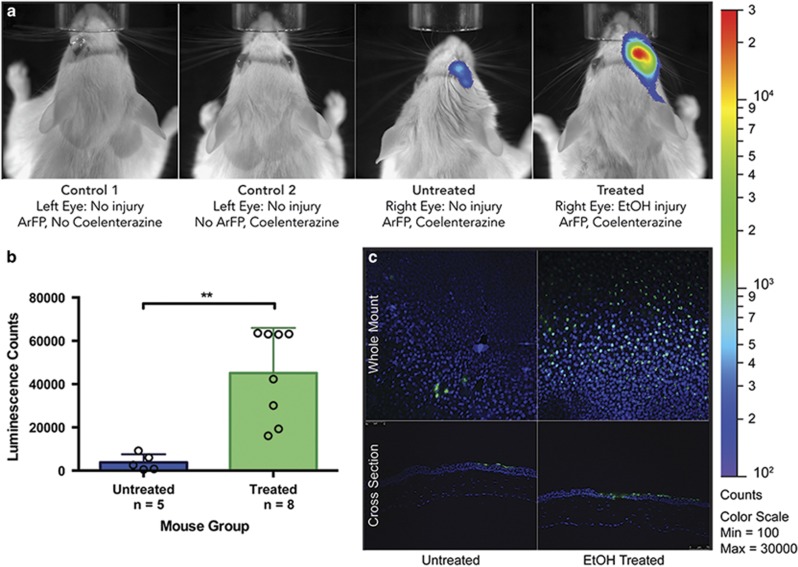
(**a**) IVIS images comparing mice from groups control 1 (addition of protein with no injury or substrate), control 2 (addition of substrate with no injury or protein), untreated (addition of protein and substrate with no injury), and treated (addition of protein and substrate following corneal injury). (**b**) Comparison of bioluminescence data from the total cohort (*n*=13) of mice receiving ethanol treatment (treated, *n*=8) or PBS (untreated, *n*=5). (**c**) TUNEL analysis of corneal sections. Whole mount (top) and cross-section (bottom) images of corneal tissues from untreated (PBS, left) and treated (ethanol, right) mice indicating the presence of apoptotic (green) and non-apoptotic (blue) nuclei

Apoptosis has an important role in numerous diseases ranging from cancer, to cardiovascular disease, to degenerative diseases such as AMD. Thus, the ability to detect and image apoptosis not just in cell culture, but also in tissues composed of heterogeneous populations of cells is highly desired. In our work, we began by investigating vascular apoptosis by employing a rat model of arterial injury, as well as corneal and retinal apoptosis models in mice. Specifically, in our first animal study, we performed balloon angioplasty on Sprague–Dawley rats to create conditions of ischemia/reperfusion in the iliac arteries (Figure 5a). Upon exposure of our ArFP reagent to the vessels, a significantly increased (*P*<0.01) bioluminescence signal was obtained from the angioplasty-treated artery (right iliac, Figure 6b, teal) when compared to either the ischemia-only (left iliac, Figure 5b, purple) or control (carotid, Figure 5b, blue) arteries. This suggests an elevated level of apoptosis from balloon angioplasty and reperfusion over ischemia/reperfusion as expected. The presence of apoptosis in treated vessels was confirmed through bioluminescence imaging with IVIS (Figure 5c), and histological analysis (Figure 5d). These results indicated that the fusion protein successfully binds to apoptotic cells in whole tissues as well. The low level of background observed in the carotid artery (Figure 5b, blue) is likely due to the known autoluminescence of coelenterazine in biological samples,^35^ and can be accounted for by setting specific thresholds of bioluminescence emission.

### Apoptosis detection *in vivo* and in disease model

In addition to examining vascular apoptosis, we also investigated the feasibility of employing our apoptosis detection technology in both corneal and retinal tissues of mice *in vivo*. We employed a mouse model of ethanol-induced corneal apoptosis in which we compared EtOH-treated and PBS-control mice following topical application of our ArFP reagent and its coelenterazine substrate (Figure 6a).^36, 37^ Controls for this experiment included topical application of ArFP or coelenterazine alone to the cornea of the animal (Figure 6a, control 1 and control 2, respectively). Both controls showed that either ArFP or the coelenterazine alone were insufficient to generate a detectable bioluminescence signal. In contrast, the application of ArFP and substrate in an eye lacking apoptosis induction (Figure 6a, untreated) resulted in a small, yet detectable signal of very low intensity, likely due to some non-specific binding of the protein to the cornea. However, when applied together to an apoptosis-induced cornea, a significantly increased (*P*<0.01) bioluminescence signal was generated (Figure 6a, treated and Figure 6b). We confirmed that the observed increase in bioluminescence corresponds to a dramatic increase in apoptotic cells by using the TUNEL analysis of corneal whole mount and cross sections from these same mice (fluorescein-positive/green) (Figure 6c). These results indicate that ArFP is capable of specifically detecting apoptosis in the corneal tissues of mice and represents the first use of bioluminescence-based *in vivo* Annexin V apoptosis detection.

**Figure 7:**
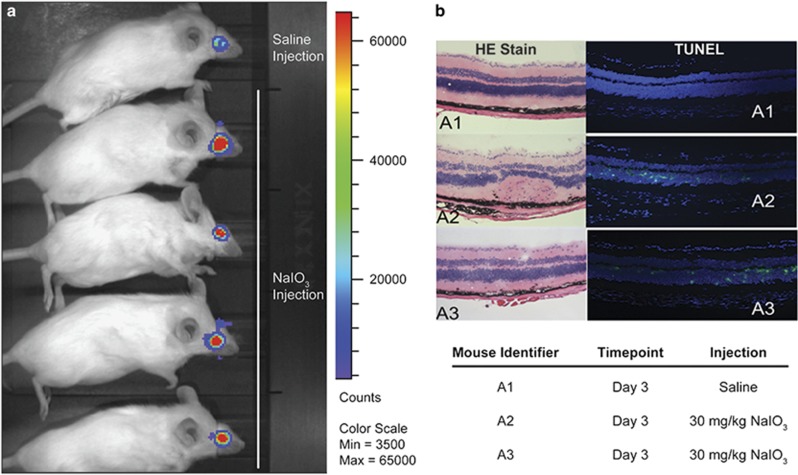
(**a**) IVIS analysis of bioluminescence comparing control (saline injection) and retinal degeneration model (NaIO_3_ injection) mice. (**b**) Histological analysis of retinal sections from control (a1, PBS injection) and retinal degeneration model (a2 and a3, NaIO_3_ injection) mice. Left: H&E stain of retinal cross sections. Right: TUNEL analysis of retinal cross sections indicating presence of apoptotic (green) and non-apoptotic (blue) nuclei

The demonstrated success of ArFP in the bioluminescence-based detection of apoptosis in corneal tissues led us to investigate further its ability to detect disease-relevant cell apoptosis and death in deeper tissues, such as the retina. It has been reported that intravenous sodium iodate (NaIO_3_) injection causes retinal degeneration, resulting in a model of AMD.^38, 39, 40, 41^ Specifically, mechanisms of apoptosis have been implicated as the underlying cause of this degeneration, and could thus be identified using the ArFP construct.^40, 41^ As shown, a strongly positive bioluminescence signal was observed from ArFP applied to the eyes of NaIO_3_ treated mice, whereas only a limited amount of background is observed for the control mouse receiving only PBS (Figure 7A). These results were verified through histological analysis of retinal cross sections, revealing a large number of apoptotic cells in the NaIO_3_-injected mice (Figure 7B, a2 and a3) that are completely absent in the saline control mouse (Figure 7B, a1).

## Discussion

In this work, we report a new bioluminescence-based Annexin V biosensor for the detection of apoptosis. Because of its involvement in both natural cell clearance within multicellular organisms as well as in numerous disease states, the ability to detect and monitor apoptosis is critically important. In addition to the identification of excessive or abnormal cell death, apoptosis monitoring can also be applied to the development and screening of numerous therapeutic agents such as those developed to induce selective cell death within tumors.

Since its identification over 30 years ago, Annexin V has been coupled with diverse reporters for the identification of apoptotic cells via recognition and binding of phosphatidylserine. To date, the vast majority of these reporters have relied on either isotopic markers or conjugation to fluorophores. As previously discussed, the chimeric nature of the ArFP construct eliminates the challenges associated with batch-to-batch variability presented by earlier platforms reported in the literature, whereas also taking advantage of the inherently higher sensitivity and lower background of bioluminescence-based detection. The analysis of the structural, physical, and biochemical properties of our bioluminescent biosensing system demonstrates that the ArFP fully retains the properties of both Annexin V and RLuc8 with no need for post-translational modification.

Importantly, we demonstrated the feasibility of using ArFP for the first time as a tool for the design and development of a novel class of bioluminescence-based Annexin V biosensors for *in vivo* detection of apoptosis. Using three different animal models, namely, rat vascular, mouse corneal, and mouse retinal apoptosis, we validated our *in vitro* detection of apoptosis, and showed successful bioluminescence imaging of disease-relevant of apoptosis. This further demonstrates that our ArFP biosensing detection method can be applied to the monitoring and study of different diseases. We envision that our ArFP biosensing system can be employed in a diverse range of clinically relevant diseases encompassing AMD, cancer, and even atherosclerosis and cardiovascular disease. In addition, unique fusion proteins between Annexin V and other bioluminescence proteins such as aequorin, obelin, *Gaussia* luciferase, and so on, can be created, enhancing the toolbox of sensing proteins with tailored characteristics, such as varied emission wavelengths, kinetics, and bioluminescence half-lives. The ability to prepare a series of fusion proteins endowed with specific characteristics allows for the design of a new class of biosensors for a variety of targeted applications. We foresee that these biosensors will find numerous applications in both *in vitro* as well as *in vivo* identification, therapeutic screening, and risk assessment of diseases involving apoptosis.

## Materials and methods

### Reagents and kits

LB broth (Miller), LB Agar (Miller), dimethylsulfoxide (DMSO), KOD hot start master mix, pET-30 Xa/LIC Vector Kit, and ApopTag Peroxidase *In Situ* Oligo Ligation Kit were purchased from EMD Millipore (Billerica, MA, USA). Kanamycin sulfate, ampicillin (sodium salt), and potassium chloride were obtained from Amresco (Solon, OH, USA). From VWR International (West Chester, PA, USA), sodium chloride, ammonium sulfate, methanol, and 4-(2-hydroxymethyl)-1-piperazine-1-ethanesulfonic acid (HEPES) were purchased. Calcium chloride, magnesium chloride, sodium iodate, l-arabinose, imidazole, heat inactivated fetal bovine serum (FBS), staurosporine, *β*-mercaptoethanol, Tween 20, paraformaldehyde, nucleotide primers, the Roche *In Situ* Cell Death Detection (TUNEL) kit, as well as mono- and di-basic phosphate salts of both potassium and sodium were all purchased from Sigma-Aldrich (St. Louis, MO, USA). Triton X-100 was purchased from Alfa Aesar (Haverhill, MA, USA). Sodium citrate was purchased from Fisher Scientific (Pittsburgh, PA, USA), whereas normal saline solution was purchased from G Biosciences (St. Louis, MO, USA). Problock Gold Bacterial 2D Protease Inhibitor Cocktail and isopropyl *β*-d-1-thiogalactopyranoside (IPTG) were purchased from Gold Biotechnology (St. Louis, MO, USA). 3-(N-morpholino)propanesulfonic acid (MOPS) and the Pierce BCA Protein Assay Kit were obtained from Thermo/Life Technologies (Rockford, IL, USA). Lipids POPC (16:0-18:1 PC, 1-palmitoyl-2-oleoyl-*sn*-glycero-3-phosphocholine) and POPS (16:0-18:1 PS, 1-palmitoyl-2-oleoyl-*sn*-glycero-3-[phospho-l-serine]) were purchased as chloroform solutions from Avanti Polar Lipids (Alabaster, AL, USA). Both high glucose Dulbeccos’ Modified Eagle Medium (DMEM) and Roswell Park Memorial Institute (RPMI) 1640 medium were purchased from Gibco/Life Technologies (Grand Island, NY, USA), whereas trypsin-versene (EDTA) was purchased from Lonza (Basel, Switzerland), antibiotic-antimycotic solution was obtained from Cellgro/Corning (Manassas, VA, USA), and Accutase cell detachment solution was purchased from Innovative Cell Technologies (San Diego, CA, USA). The CellTiter 96 AQ_ueous_ One Solution Cell Proliferation Assay kit was obtained from Promega (Madison, WI, USA). Primary antibodies were purchased from Abcam (Cambridge, MA, USA) and Cell Signaling Technology (Danvers, MA, USA), whereas native coelenterazine was purchased from Nanolight Technology (Pinetop, AZ, USA). The pan-caspase inhibitor Z-VAD(OMe)-FMK and the Annexin V-FITC Early Apoptosis Detection Kit were also purchased from Cell Signaling Technology. Competent NEB5*α* and T7 Express *Escherichia coli* strains were obtained from New England Biosciences (Ipswich, MA, USA). Ni-NTA agarose and the QIAprep Spin Miniprep Kit were purchased from Qiagen (Hilden, Germany). Laemmli sample buffer and Mini-Protean TGX (4–20%) gels were obtained from Bio-Rad (Hercules, CA, USA). Odyssey Blocking Buffer (PBS) and secondary antibodies were purchased from LI-COR Biosciences (Lincoln, NE, USA). Isoflurane, USP was purchased from Piramal Healthcare (Bethlehem, PA, USA), and VECTASHIELD Antifade Mounting Medium with DAPI was purchased from Vector Laboratories (Burlingame, CA, USA).

### Fusion gene design and transformation

Plasmid pPROEX HTb containing Annexin V was provided by Prof. Seamus Martin, Trinity College, Dublin, Ireland. The Annexin V gene was isolated using forward (**GGTATTGAGGGTCGC**ATGGCACAGGTTCTC) and reverse (AGAACCACCAGAACCACCGTCATCTTCTCCACA) primers with appropriate regions for inclusion into the pET-30 Xa/LIC vector (bolded) as well as for use in overlap-extension PCR (OE-PCR, underlined). The RLuc8 gene was isolated from pBAD/Myc-HIS A::RLuc8 using similarly designed forward (GGTGGTTCTGGTGGTTCTATGGCTTCCAAGGTG) and reverse (**AGAGGAGAGTTAGAGCC**CTGCTCGTTCTTCAG) primers. Following fusion gene generation via OE-PCR, cloning was performed as per the pET-30 Xa/LIC Vector Kit’s instructions, and the product was transformed into competent NEB5*α* and T7 Express *E. coli*. Nucleotide sequencing analysis performed on plasmids isolated from colonies of these bacteria confirmed the presence of a fusion gene consisting of Annexin V and RLuc8, henceforth referred to as pET-30/ArFP (Supplementary Figure S2).

### Protein expression

Five milliliters Growth Media (LB Broth (Miller) with 35 *μ*g/ml kanamycin) was inoculated with a T7 Express bacterial colony and grown overnight at 37 °C/250 r.p.m. After this growth period, 300 ml Growth Media was inoculated with 1 ml of this overnight culture and grown at 37 °C/250 r.p.m. to an OD_600_ of 0.6. Protein expression was induced via addition of IPTG to a final concentration of 1 mM, and expression continued for 2 hours at 37 °C/250 r.p.m. Cells were collected via centrifugation at 5000x*g* for 25 min at 4 °C, and the pellet was resuspended in 10 ml Lysis Buffer (50 mM sodium phosphate, 300 mM sodium chloride, 10 mM imidazole, pH=8) and 100 *μ*l Problock Gold Bacterial Protease Inhibitor Cocktail was added. Purification of the ArFP construct was achieved utilizing an encoded N-terminal 6 × Histidine tag as described by Qiagen. Expression and purification of native Annexin V and RLuc8 was achieved using this protocol with minor changes: both were expressed in Amp Growth Media (LB Broth (Miller) with 0.1 mg/ml ampicillin), and expression of RLuc8 was induced via addition of 0.2% arabinose.

### Verification and characterization of ArFP

#### Western blot analysis

A total of 1 *μ*g purified protein was mixed with 2x Laemmli Sample Buffer with 5% *β*-mercaptoethanol, resolved by SDS-PAGE, and transferred to nitrocellulose membranes for blotting. Either anti-Annexin V mouse monoclonal (ab54775) or anti-*Renilla* luciferase rabbit polyclonal (ab187338) antibody were used as indicated, and membranes were imaged using the LI-COR Odyssey Classic (Model 9120) Imaging System (LI-COR Biosciences, Lincoln, NE, USA).

#### Bioluminescence measurement and characterization

Purified ArFP and RLuc8 were dialyzed into ArFP binding buffer (10 mM HEPES, 100 mM NaCl, 5 mM KCl, 1.8 mM CaCl_2_, 1 mM MgCl_2_, pH=7.4), and an aliquot of 100 *μ*l was added in triplicate to a 96 well plate at concentrations ranging from 50 pM to 500 nM. Bioluminescence intensities were recorded using a PolarSTAR Optima (BMG LABTECH GmbH, Ortenberg, Germany) following injection of 2.5 *μ*g/ml coelenterazine. Decay kinetics were measured by integrating the bioluminescence emission signal from the 500 nM sample in 0.24 s intervals for 50 s (coelenterazine injection occurred at 10 s). Bioluminescence spectra were obtained for both proteins using a Luminoskan Ascent Microplate Luminometer, (Thermo Fisher Scientific, Waltham, MA, USA) recorded from 400 nm to 700 nm.

#### CD spectroscopy analysis

Samples of ArFP, Annexin V, and RLuc8 were dialyzed into CD Buffer (10 mM potassium phosphate, 100 mM ammonium sulfate, pH=7.4) at a concentration of 0.1 mg/ml, and the far-UV CD spectra were recorded using a Jasco J-815 Spectropolarimeter (Jasco, Easton, MD, USA) (scan mode: continuous; scan speed: 50 nm/min; data pitch: 0.5 nm; bandwidth: 1 nm; data integration time (D.I.T.): 2 s; accumulations: 3).^42^ Analysis of the ArFP CD data was performed using Dichroweb (http://dichroweb.cryst.bbk.ac.uk, Department of Crystallography, Institute of Structural and Molecular Biology, Birkbeck College, University of London, UK),^43^ and secondary structure assignments were generated using the CDSSTR analysis package (utilizing reference sets 3, 4, 6, 7, SMP 180, and SP 175).^44, 45^ Thermostability analysis was measured as the ellipticity at 222 nm as a function of temperature from 25 to 90 °C (scan speed: 1 °C/min; bandwidth: 2 nm; D.I.T: 2 s).^46^

#### Isothermal titration calorimetry

ITC was performed using a Nano ITC (TA Instruments, New Castle, DE, USA) and a modified protocol for the characterization of Annexin V.^31^ ArFP was dialyzed into ITC Buffer (20 mM MOPS, 100 mM KCl, 0.75 mM CaCl_2_, pH=7.5) to a final concentration of 0.029 mM, whereas vesicles composed of 60:40 POPC:POPS were prepared in a sample of the final dialysate to a concentration of 30 mM (protocols for generation and sizing of these lipid vesicles found in Supplementary Figure S4). Titration of vesicles into ArFP solution was performed via 24 × 9.14 *μ*l injections, and the integrated heat signals were analyzed with the NanoAnalyze Software (v 2.3.6, TA Instruments) using an independent binding model.

### Cell culture

Human T lymphocyte interleukin-2 (Jurkat, Clone E6) cells (ATCC TIB-152) were grown in RPMI 1640 supplemented with 10% FBS, 100 mg/l penicillin, 100 mg/l streptomycin, and 2 mM glutamine.

#### Apoptosis induction

Cells were grown in Corning T25 Tissue Culture Flasks to an approximate density of 5 × 10^6^ cells/ml. Apoptosis was induced via addition of 1 *μ*M of the known inducer staurosporine.^47^ For apoptosis inhibition studies, the synthetic peptide Z-VAD(OMe)-FMK was added to the cells at a final concentration of 25 *μ*M 1 h prior to the induction of apoptosis via staurosporine. For bioluminescence-based detection of apoptosis, cells were collected following incubation, centrifuged, and resuspended in binding buffer containing 1 nM ArFP for 5 min. Cells were then washed three times with binding buffer, and an equal number of cells were transferred to a 96 well plate in triplicate for bioluminescence measurements.

#### Flow cytometric analysis of apoptosis

After induction of apoptosis, cells from each treatment condition were washed once in PBS and resuspended in Annexin binding buffer and stained with FITC-conjugated Annexin V and PI as per the manufacturer’s instructions. After staining, cells were then washed with Annexin binding buffer, resuspended in a fixing solution of PBS containing 4% PFA, and stored at 4 °C in the dark. Flow cytometry of samples was performed using a CytoFLEX S (Beckman Coulter Life Sciences, Indianapolis, IN, USA). Annexin-FITC fluorescence (FL1) was collected through a 525/40 band pass filter, whereas PI fluorescence (FL11) was collected through a 610/20 band pass filter. Data acquisition (2 × 10^4^ events per sample) was performed using the CytExpert 1.2 software (Beckman Coulter Life Sciences).

#### Western blot analysis of caspase activation

Cells treated with staurosporine (or control) were collected (~5 × 10^7^ total cells per condition) and washed with PBS. Cells were lysed via addition of 1 ml ice-cold RIPA buffer containing 10 *μ*l Halt protease and phosphatase inhibitor (100 ×), and were stored at 4 °C for 30 min. Lysates were clarified via centrifugation at 12 000x*g* for 25 min, and 1 *μ*g of total protein (cell lysate) from each condition was analyzed via western blot as previously described using anti-pro-caspase-3 rabbit polyclonal (CST #9662) and anti-*β*-actin mouse monoclonal (CST #3700) antibodies.

### Animal models of apoptosis

All animal studies performed within this work were carried out in accordance with the recommendations in the Guide for the Care and Use of Laboratory Animals of the National Institutes of Health and were based on protocols approved by the Institutional Animal Care and Use Committee (IACUC) at the University of Miami.

#### *Ex vivo* rat iliac artery apoptosis induction

Three male Sprague–Dawley rats (12 weeks old, 280–320 g, Harlan, Indianapolis, IN, USA) were used to induce vascular apoptosis in the right iliac artery via balloon injury as previously described.^48^ Briefly, the abdominal and iliac arteries were clamped at proximal and distal sites, respectively, and a 2 Fr. Fogarty Arterial Embolectomy Catheter with a 4 mm balloon (Edwards Lifesciences, Irvine, CA, USA) was directed to the right iliac to induce injury. Blood flow was restored, and 10% BSA was used to reduce non-specific binding. Following washing with Binding buffer, 100 *μ*l 2 nM ArFP solution was added directly to the vessel surface, and incubated for 3 min. The vessels were again washed with binding buffer, and the right iliac (treated), left iliac (untreated), and carotid (control) arteries were excised and stored in binding buffer. Bioluminescence measurements were performed via addition of 50 *μ*l of 0.1 mg/ml coelenterazine and recorded using either a Turner BioSystems 20/20n Luminometer (Promega, Madison, WI, USA), or a Caliper/Xenogen IVIS SPECTRUM *in vivo* imaging system (IVIS, Caliper, Hopkinton, MA, USA). Immunohistochemical staining of balloon-injured (or control) arteries collected 30 min after treatment were either visualized with hematoxylin and eosin (H&E) stain, or used for apoptosis detection with the ApopTag Peroxidase *In Situ* Oligo Ligation Kit (EMD Millipore) according to the manufacturer’s guidelines. Apoptotic cells were identified by a dark brown nucleus surrounded by a red cytoplasm.

#### *In vivo* mouse corneal apoptosis detection

Corneal injury was induced in the right eye of eight female BALB/c mice (8 weeks old, Charles River, Boston, MA, USA) via exposure to 20% ethanol for 40 s following anesthetization via ketamine/xylene (1.5 mg/0.3 mg) injection.^36, 49^ The left eyes of these mice were treated with PBS as internal controls, after which both eyes were rinsed with normal saline. Five additional mice were used as untreated controls, receiving PBS on both eyes. 4 h post-treatment, 10 *μ*l 2 nM ArFP solution was added to the examined eye (treated or control) for 1 min. Eyes were washed with binding buffer, and bioluminescence was recorded via addition of 5 *μ*L of 0.1 mg/ml coelenterazine and imaged using IVIS. Whole mount and cross-section corneal samples were fixed using 2% paraformaldehyde, and apoptosis was visualized using the terminal deoxynucleotidyltransferase (TdT) dUTP-biotin nick-end labeling (TUNEL) kit (*In Situ* Cell Death Detection Kit, Roche, Basel, Switzerland) according to the manufacturer’s protocol. Images of mounted samples were obtained with a Zeiss confocal microscope exciting at 488 nm for fluorescein (green) and 405 nm for DAPI (blue).

#### *In vivo* mouse retina apoptosis detection

Following anesthetization with ketamine/xylene (1.5 mg/0.3 mg), four female BALB/c mice (8 weeks old, Charles River) received tail-vein injection of 30 mg/kg sodium iodate (NaIO_3_), whereas a control mouse received a volume matched injection of PBS. Three days following injection, 10 *μ*l 2 nM ArFP solution was added topically to the right eye of each mouse for 1 min, and washed with binding buffer. Bioluminescence imaging performed via addition of 5 *μ*l of 0.1 mg/ml coelenterazine followed immediately by image capture via IVIS. Retinal samples from an additional 3 mice (two NaIO_3_-treated, one PBS-control) were prepared in a cryomold and sectioned for histological analysis. Sections were then either stained using H&E or treated with the TUNEL assay kit for apoptosis detection as previously described.

#### Statistical analyses of animal studies

For purposes of *in vivo* apoptosis assessment, the number of animals was limited to avoid unnecessary use of living subjects. Although power analysis was not performed prior to data acquisition, the number of animals selected (*n*=3 for vascular apoptosis and *n*=13 for corneal apoptosis) was sufficient to generate a statistically relevant difference in values between populations. Data are given as mean and standard deviations, whereas data points for each individual are shown for all statistical analyses of the animal studies. Obtained data were assumed normally distributed, and statistical significance of the collected data was determined with the student’s *t-*test (unpaired, two-tailed) using the analysis software Prism 6.0 (GraphPad Software, La Jolla, CA, USA). Healthy individuals were used for all experiments with no requisite randomization. Animals were neither selected for nor against additional specific criteria, and researchers were not blinded to these selections.

## Figures and Tables

**Figure 1:**
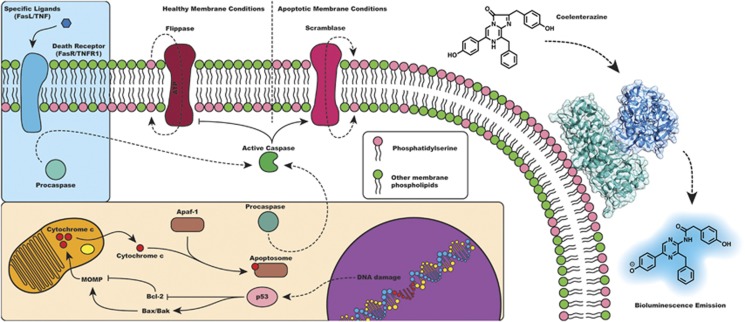
Pathways of apoptosis (extrinsic – blue box and intrinsic – orange box) and detection concept. Both pathways converge to the activation of caspases that are principally responsible for morphological and biochemical changes associated with apoptosis including PS exposure. Although PS is sequestered to the inner-face of the plasma membrane under healthy conditions, the ArFP construct exhibits the ability to bind PS at surface of apoptotic cells and generate a bioluminescent signal in the presence of substrate coelenterazine

**Table 1:** Summary of protein characteristics

	**Calculated molecular weight (kDa)**^a^	**Binding affinity for PS (K_D_, μM)**	**Maximum bioluminescence emision (nm)**	**Bioluminescence half-life (s)**
Annexin V	39.3	13 (31)	—	—
RLuc8	39.5	—	487 (28)	3.11 (28)
ArFP	77.5	20.7	492	3.59

a Molecular weights determined from primary amino acid sequence
