# Supplementary material for: An enhanced bioluminescence-based Annexin V probe for apoptosis detection in vitro and in vivo
Source: Cell Death Dis. 2017 May 25;8(5):e2826–. doi: 10.1038/cddis.2017.141 (PMC5520691; doi:10.1038/cddis.2017.141)
Supplement: Supplementary Information [file cddis2017141x1.docx]

**An enhanced bioluminescence-based Annexin V probe for apoptosis detection *in vitro* and *in vivo***

Trajen Head^1^, Peter Dau^1^, Stephanie Duffort^2^, Pirouz Daftarian^3^, Pratibha Joshi^1^, Roberto Vazquez-Padron^4^, Sapna K Deo^1^, Sylvia Daunert^1^

^1^ Department of Biochemistry and Molecular Biology, University of Miami Miller School of Medicine, Miami, FL 33136, USA

^2^Department of Ophthalmology, University of Miami Miller School of Medicine, Miami, FL 33136, USA

^3^NGM Biopharmaceuticals, Inc., South San Francisco, CA 94080, USA

^4^Department of Surgery, University of Miami Miller School of Medicine, Miami, FL 33136, USA

Correspondence: Sylvia Daunert, Department of Biochemistry and Molecular Biology, University of Miami Miller School of Medicine, 1011 NW 15^th^ Street, Gautier Building Room 239DA, Miami, FL 33136. Tel: (305) 243-6282; Fax: (305) 243-3995; E-mail: SDaunert@miami.edu

**Supplemental Information**

**Supplemental Figure S1:** Structure of Annexin V and RLuc8 and mechanism of bioluminescence emission from substrate coelenterazine


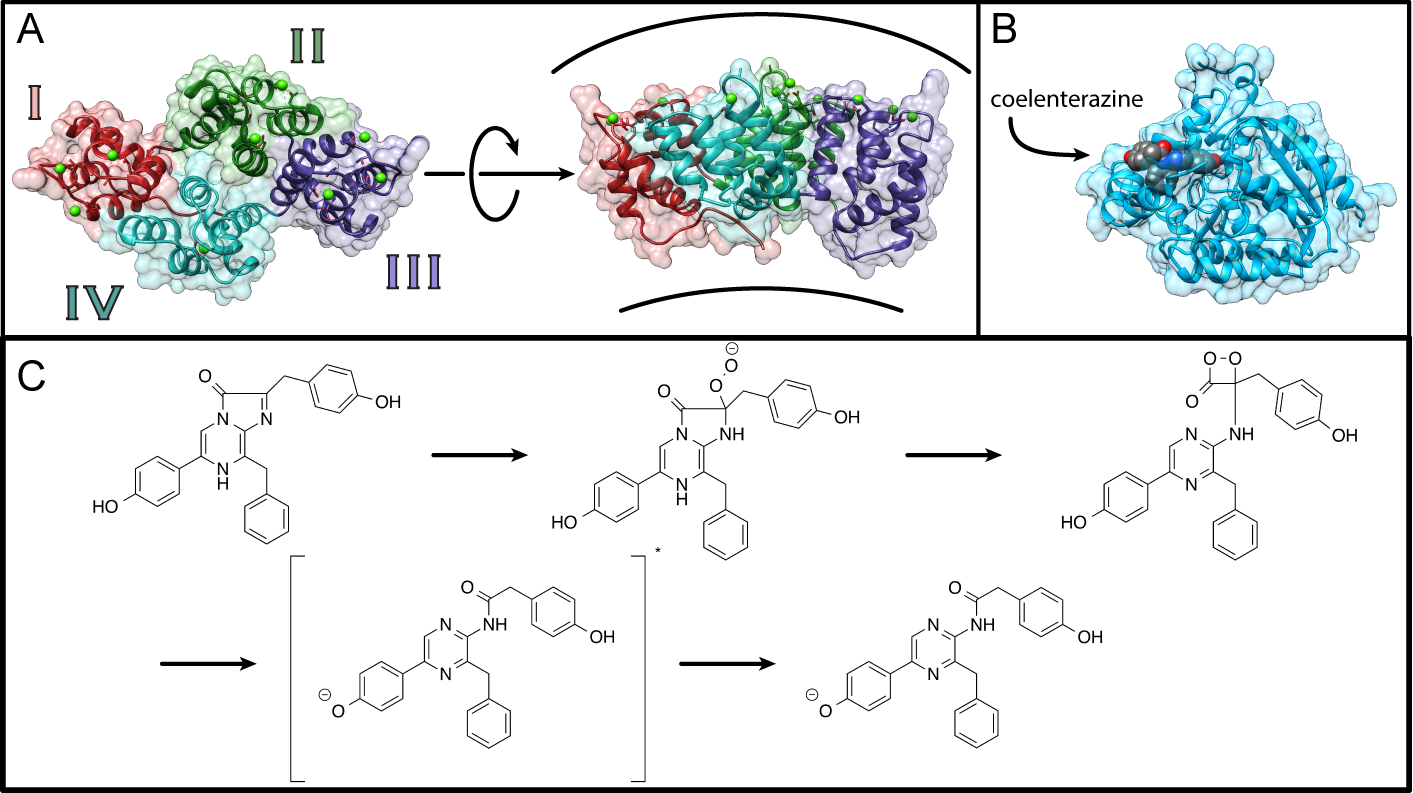


Figure S1: A) Structure of Annexin V (PDB accession number 1ANX) with four repeated annexin core domains colored individually. The convex face of the protein binds PS at the surface of apoptotic cells. B) Structure of *Renilla* luciferase with bound substrate coelenterazine (PDB accession number 2PSJ). C) Oxidative decarboxylation of RLuc8 substrate coelenterazine.

**Supplemental Figure S2:** Cloning of component genes and generation of ArFP Fusion Gene


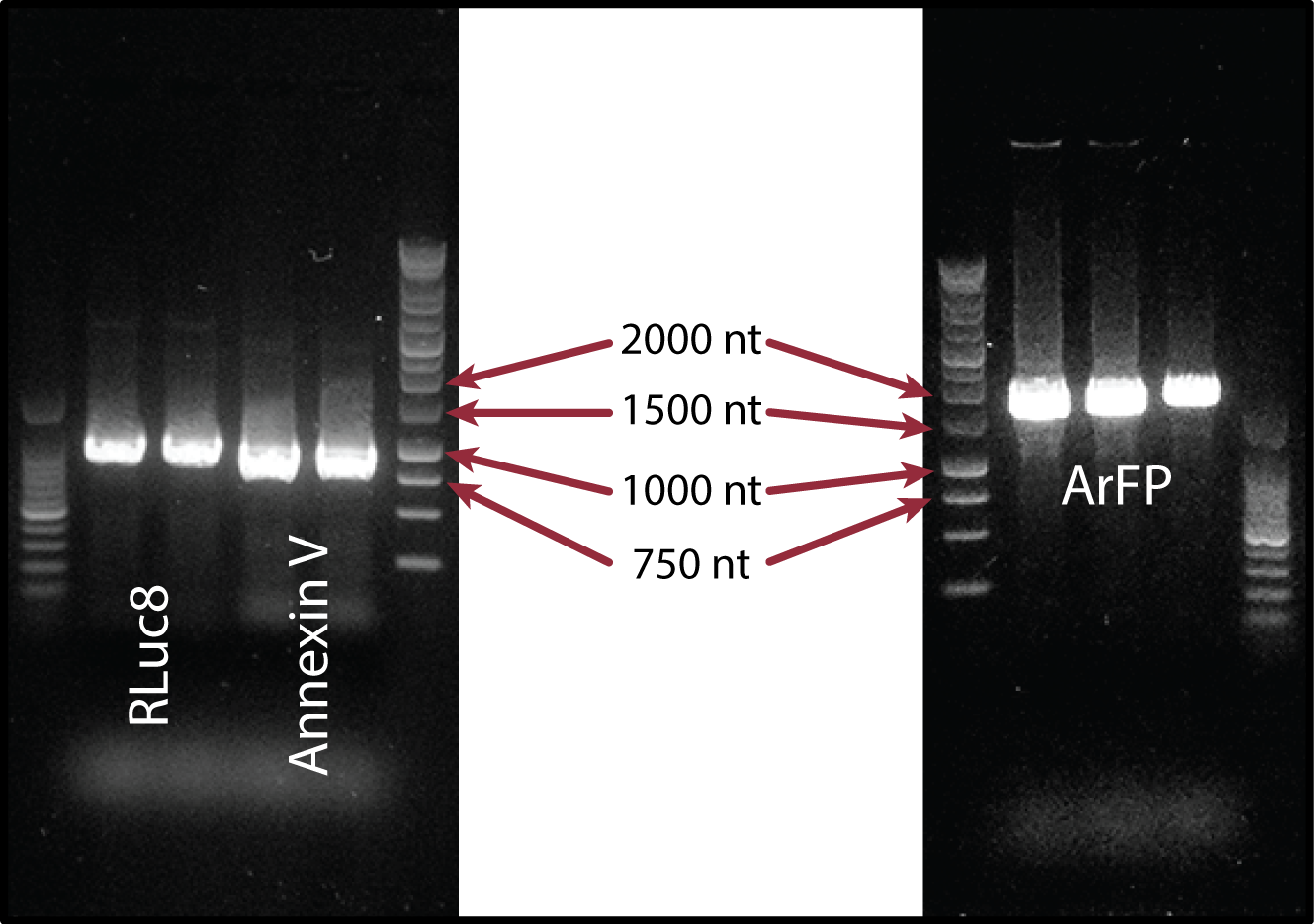


Figure S2: Left) Isolation of genes for RLuc8 and Annexin V from plasmids pBAD/Myc-HIS A::RLuc8 and pPROEX HTb, respectively. Component genes exhibit sizes of approximately 990 nt for Annexin V and 1020 nt for RLuc8. Right) Isolation of fusion gene following OE-PCR, generating a single product of approximately 2000 nt

**Supplemental Figure S3:** Plasmid map of pET-30/ArFP


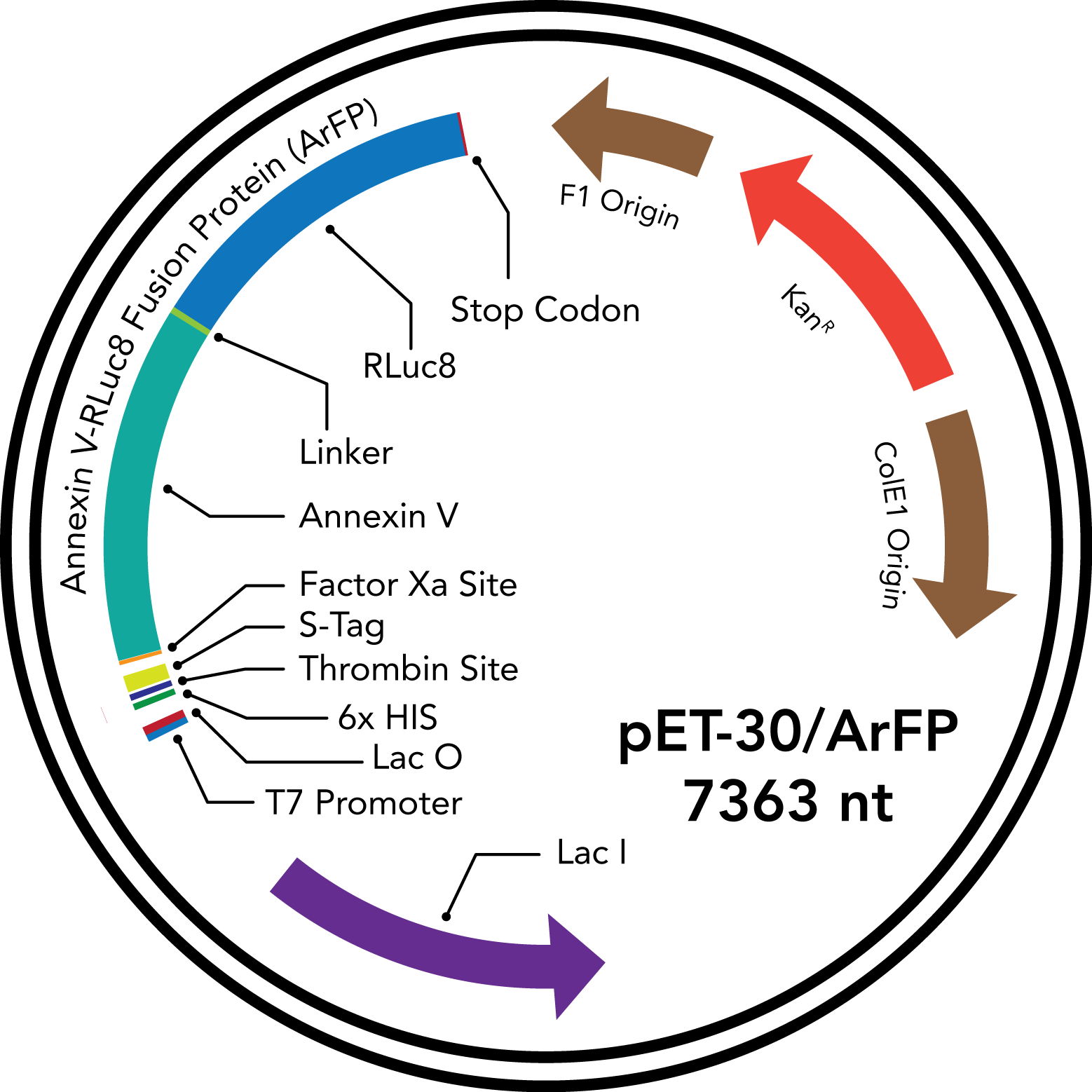


**Generation and Sizing of Lipid Vesicles**

A mixture of POPC and POPS in chloroform was prepared in a round bottom flask with a molar ratio of 60:40 POPC:POPS. The chloroform was subsequently removed via rotary evaporation. The resulting thin lipid film was rehydrated to a final concentration of 30 mM using a volume of the final dialysate used to prepare the ArFP sample. Rehydration was achieved by rotating the round bottom flask without vacuum for 1 hour. Following rehydration, the Avanti^®^ Mini-Extruder (Avanti^®^ Polar Lipids, Inc., Alabaster, AL) was used to downsize the lipid vesicles via extrusion through a 0.2 μm polycarbonate filter, and the size of these vesicles was determined via dynamic light scattering (DLS) using a Malvern Zetasizer Nano – ZS90 (Malvern, Worcestershire, United Kingdom).

**Supplemental Figure S4:** Sizing of 60:40 POPC:POPS Lipid Vesicles

**Caco-2 Cell Culture**

Caco-2 heterogeneous human epithelial colorectal adenocarcinoma cells (ATCC^®^ HTB-37^™^) were grown in DMEM, supplemented with 10% fetal bovine serum (FBS), 100 mg/L penicillin, 100 mg/L streptomycin, and 2 mM glutamine.

*Apoptosis Induction*. Cells were grown in Corning^®^ T25 Tissue Culture flasks to approximately 80 % confluence. Apoptosis was induced via addition of 1 μM of the known inducer staurosporine. After incubation with staurosporine, the media was carefully aspirated, and the cells washed with 5 mL PBS. Washed cells were then removed from the growth surface via addition of 5 mL Accutase^®^ cell detachment solution. Detached cells were collected, centrifuged, and resuspended in Binding Buffer containing 1 nM ArFP for 5 minutes. Cells were then washed three times with Binding Buffer, and an equal number of cells were transferred to a 96 well plate in triplicate for bioluminescence measurements. Time-dependent cytotoxicity of staurosporine for both Caco-2 and Jurkat cells was studied by MTS (3-(4,5-dimethylthiazol-2-yl)-5-(3-carboxymethoxyphenyl)-2-(4-sulfophenyl)-2H-tetrazolium) assay using the CellTiter 96^®^ AQ_ueous_ One Solution Cell Proliferation Assay kit (Promega, Madison, WI) according to the manufacturer’s procedure.

**Supplemental Figure S5:** *In vitro* apoptosis detection using Caco-2 cells


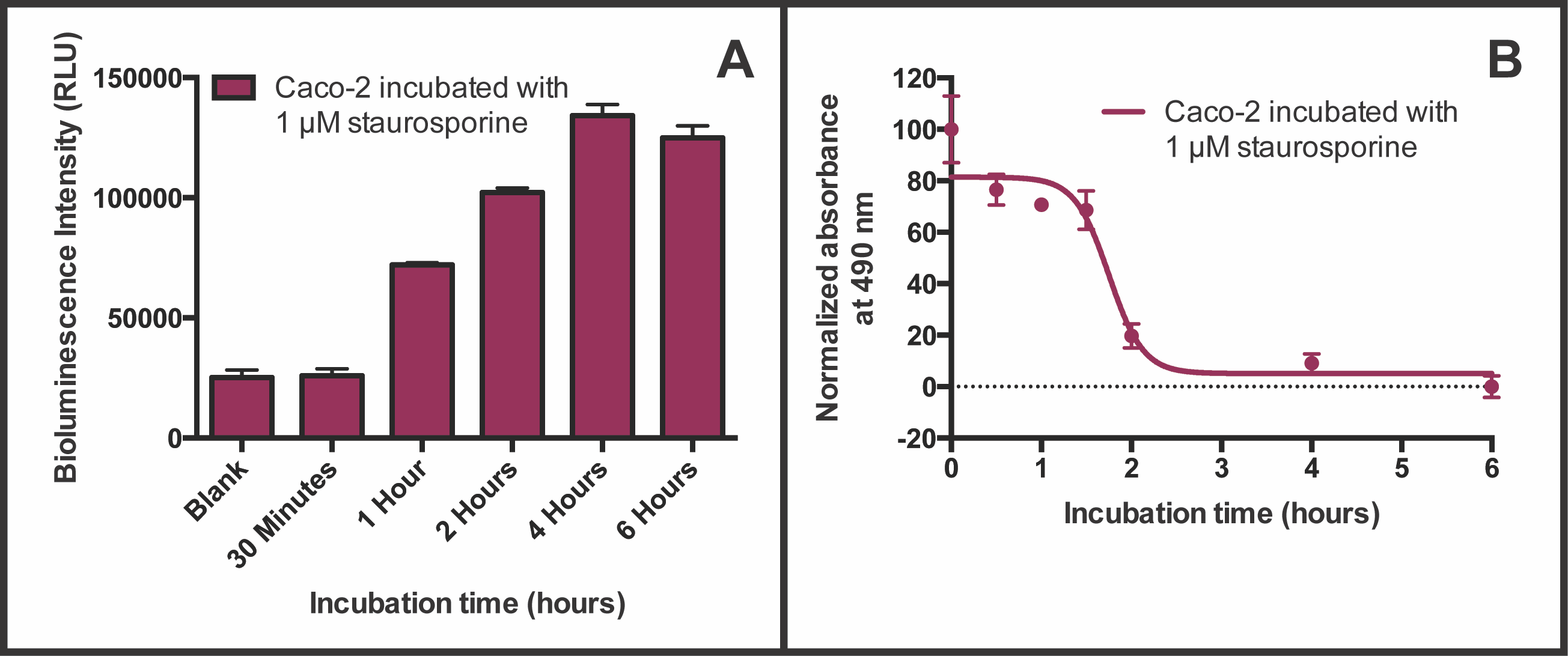


**Figure S5**: A) Bioluminescence response of ArFP incubated with Caco-2 cells after treatment with 1 μM staurosporine for various times. B) Absorbance at 490 nm of wells containing cells incubated with MTS for 4 hours following treatment with 1 μM staurosporine for various times.

**Supplemental Figure S6**: *In vitro* cell metabolic activity using Jurkat cells


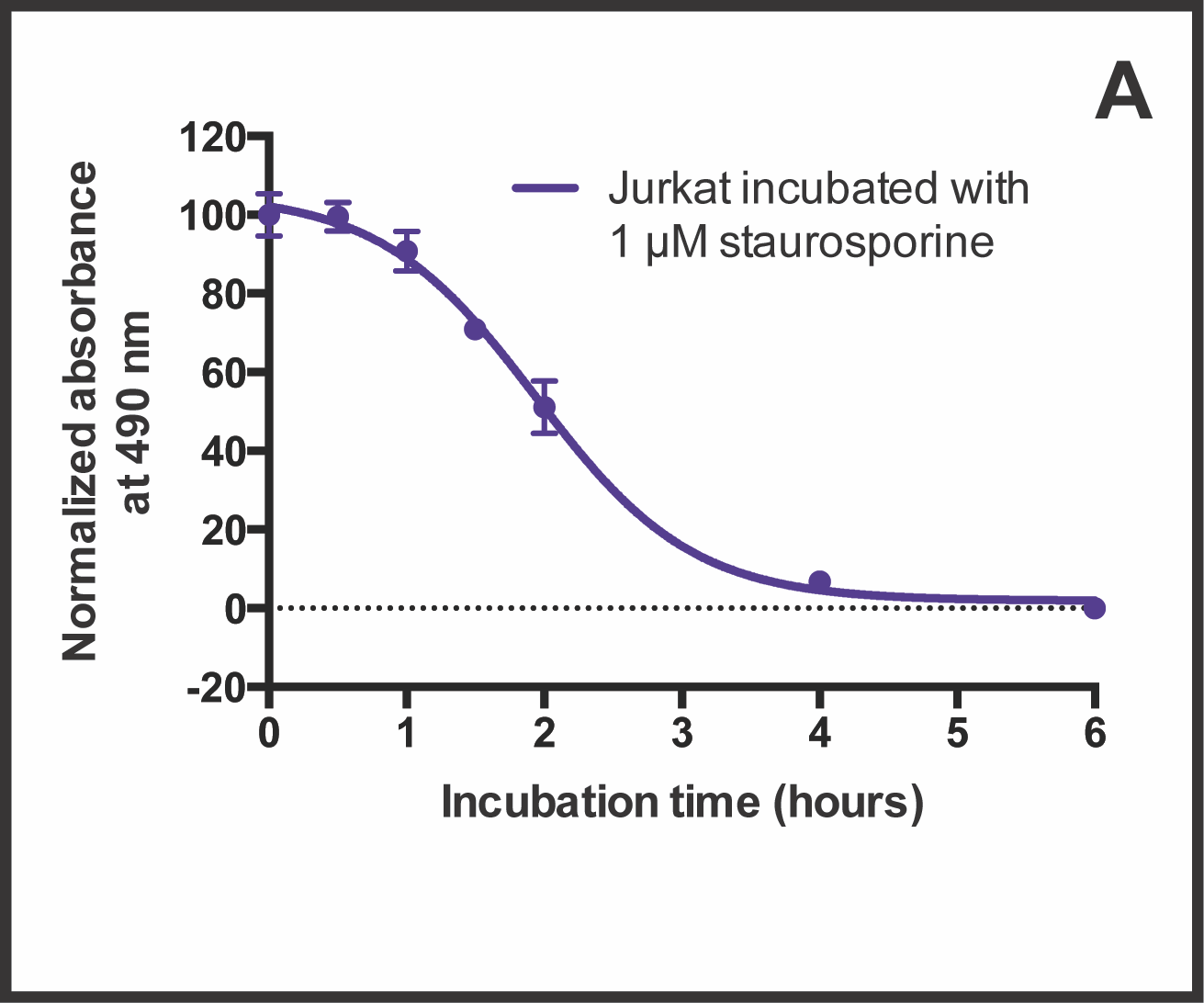


**Figure S6**: Absorbance at 490 nm of wells containing cells incubated with MTS for 4 hours following treatment with 1 μM staurosporine for various times.
